# Adjuvant therapy of severe and/or refractory bullous pemphigoid with immunoadsorption – A prospective monocenter pilot study

**DOI:** 10.1111/ddg.15909

**Published:** 2025-11-02

**Authors:** Maike M. Holtsche, Nina van Beek, Christoph M. Hammers, Artem Vorobyev, Michael Kasperkiewicz, Nina Schumacher, Philip Muck, Enno Schmidt

**Affiliations:** ^1^ Department of Dermatology University of Lübeck Lübeck Germany; ^2^ Department of Dermatology University of Oldenburg Oldenburg Germany; ^3^ Division of Dermatology Department of Medicine David Geffen School of Medicine at University of California Los Angeles Los Angeles USA; ^4^ Department of Internal Medicine University of Lübeck Lübeck Germany; ^5^ Lübeck Institute of Experimental Dermatology University of Lübeck Lübeck Germany

**Keywords:** autoimmune blistering disease, BP180, bullous pemphigoid, immunoadsorption

## Abstract

**Background and Objectives:**

Bullous pemphigoid (BP) is the most common autoimmune blistering disease in the Western world. While remission is achieved in the majority of BP patients by long‐term use of corticosteroids with or without immunomodulants/immunosuppressants, national and international guidelines recommend adjuvant immunoadsorption (IA) in refractory patients. Here we investigated the safety and efficacy of IA in severe and/or refractory BP patients.

**Patients and Methods:**

10 BP patients (3 women, 7 men; mean age 69.7 years; range 52–81 years) were treated with IA on three consecutive days (LigaSorb^®^, Fresenius Medical Care) combined with oral prednisolone (0.5 mg/kg BW per day, tapered), dapsone (1.5 mg/kg BW per day), and lesional mometasone furoate ointment.

**Results:**

Within 2 and 6 months after IA, 50% and 90% of patients showed complete remission on therapy, respectively. In all patients, serum anti‐BP180 IgG levels decreased significantly by an average of 89% and 73% immediately after the third IA and 4 weeks later, respectively. A total of 56 adverse events (AE) occurred during the 12‐month follow‐up. The majority of AE were of severity grade 2 (50%), 15 AE were classified as severe (grade 3–4).

**Conclusions:**

IA can be considered as a relatively safe and effective adjuvant therapy for patients with severe or refractory BP.

## INTRODUCTION

Autoimmune blistering diseases (AIBD) are caused by autoantibodies against structural proteins of the epidermis or dermal‐epidermal junction.^1^, ^2^ With an incidence of about 20 patients/million/year bullous pemphigoid (BP) is the most frequent AIBD in Central and Northern Europe and Northern America.^3^, ^4^, ^5^, ^6^ The target antigens in BP are two structural proteins of the dermal‐epidermal junction, BP180, also termed type XVII collagen or BPAG2, and BP230 (BPAG1).^7^ Detection of tissue‐bound IgG and complement C3 by direct immunofluorescence (IF) is the diagnostic gold standard. Circulating autoantibodies can be detected by indirect IF on human salt‐split skin as well as by ELISA with recombinant forms of the two target antigens and the indirect IF‐based BIOCHIP^®^ technique.^7^, ^8^, ^9^ The NC16A domain of BP180 was found to be the immunodominant region.^10^, ^11^ Bound autoantibodies to BP180 lead to dermal‐epidermal separation via complement activation, the release of proinflammatory mediators, and finally, specific proteases.^12^, ^13^

The current European and German S2k guidelines for the management of BP recommend treatment with topical and systemic corticosteroids, dapsone, doxycycline, methotrexate, azathioprine, mycophenolate mofetil, and omalizumab. In patients with refractory disease, adjuvant anti‐CD20 antibody rituximab, high‐dose intravenous immunoglobulins, dupilumab, and immunoadsorption (IA) are recommended.^14^, ^15^ Due to the severe and frequent side effects of systemic corticosteroids including increased mortality in the elderly patient population of BP, corticosteroid‐sparing therapies are employed. Since anti‐BP180 autoantibody levels correlate with disease activity and the pathogenic effect of anti‐BP180 IgG has clearly been shown in vitro and in vivo,^10^, ^16^, ^17^, ^18^ removal of autoantibodies from the circulation appears to be a rational treatment option.

Immunoadsorption is a hemoadsorption procedure that selectively removes antibodies from plasma. The advantage over plasmapheresis is its relative selectivity, no need for substitution with human albumin or fresh‐frozen plasma, and the two to three times higher plasma volume that can be processed per session. It is hypothesized that IA leads to a shift of the pathogenic tissue‐bound IgG autoantibodies back into the circulation.^19^ Common side effects are citrate‐induced hypocalcemia, hypotension and infections due to venous access.^20^

Immunoadsorption has already successfully been applied in the treatment of various neurological, cardiological, and rheumatological diseases as well as in the field of nephrology, hematology and transplantation medicine.^21^ Case series of successful IA application have also been reported in patients with refractory atopic dermatitis and pemphigus.^22^, ^23^, ^24^, ^25^, ^26^ Recently, a multicenter randomized controlled trial in patients with pemphigus vulgaris and foliaceus demonstrated a significantly lower cumulative prednisolone dose in the IA plus best medical treatment group compared with the group receiving best medical treatment alone. However, the primary endpoint, i.e., the time to complete remission on therapy, was not significantly different between the groups.^27^

In the present study, ten patients with severe and/or refractory BP were treated with IA in combination with standard BP therapy, i.e., oral prednisolone 0.5 mg/kg body weight (BW) per day (tapering), topical mometasone furoate ointment, and dapsone.

## PATIENTS AND METHODS

### Patients

Patients were treated with adjuvant IA between June 2018 and October 2021 in the Department of Dermatology, University of Lübeck, Germany. In the great majority of patients, IA was applied before the outbreak of the SARS‐CoV2 pandemic in Germany, only the IA of patient 10 fell within the pandemic period. Four patients with severe BP and six which were refractory to first‐line BP therapy were included. The group of refractory patients received previous medication with prednisolone, dapsone, i.v. dexamethasone pulses, and azathioprine as detailed in Table 1. According to the guidelines of the *German Society of Dermatology*, severe disease was defined as more than 30% of the body surface area.^15^ Diagnosis was based on positive direct IF and circulating anti‐BP180 IgG by ELISA (Euroimmun, Lübeck, Germany).^11^ Exclusion criteria were acute and chronic infections, coagulation disorders, severe cardiovascular diseases, active malignancy, use of ACE inhibitors, pregnancy, and breastfeeding as well as allergies to study medication. The study was performed according to the *Declaration of Helsinki* and approved by the ethics committee of the University of Lübeck (17‐185). Three women and seven men were included after written consent. The mean age was 69.7 years ranging from 52–81 years. Patients suffered from a severe reduction in quality of life with a mean DLQI (Dermatology Life Quality Index) of 19.1/30 (2–27) at the time of inclusion (Table 1). Most patients were at that time in good general condition as measured by ECOG‐Index (Index of Eastern Co‐operative of Oncology Group) (Table 1).

**TABLE 1:** Patients’ characteristics at baseline and previous treatments.

Patient no.	Age (years)	Sex	Disease activity	Disease duration (months)	Previous treatments	ECOG	DLQI
1	72	F	Severe	0	None	2	12
2	81	M	Severe	0	None	1	13
3	79	F	Refractory	3	PRE, DAP, DEX	1	14
4	65	M	Refractory	4	PRE, DAP, DEX	1	27
5	74	M	Refractory	2	PRE, DAP	3	21
6	57	F	Severe	0	None	1	27
7	80	M	Severe	0	None	1	2
8	58	M	Refractory	60	AZA, DEX, DAP, PRE	1	26
9	79	M	Refractory	14	PRE, DAP	0	23
10	52	M	Refractory	60	PRE, DAP	0	26

*Abbr*.: AZA, azathioprine; DAP, dapsone; DEX, dexamethasone; DLQI, Dermatology Life Quality Index; ECOG, Eastern Cooperative Oncology Group; PRE, prednisolone

### Treatment protocol

Immunoadsorption was performed at the Department of Nephrology, Department of Internal Medicine I, University of Lübeck. In all patients, venous access was established via a temporary, non‐tunneled central venous dialysis catheter (Shaldon) placed in the internal jugular vein, and plasma separation was carried out using a plasma separation device (COM.TEC^®^, Fresenius Kabi AG, Bad Homburg, Germany). For anticoagulation, patients received 5,000 IU of heparin sodium as well as citrate dextrose, formula A (ACD‐A, Fresenius HemoCare Austria GmbH, Eugendorf, Austria). Plasma was passed through a protein A column (Ligasorb^®^, Fresenius Medical Care, Bad Homburg, Germany) using a plasma flow monitor (ADAsorb^®^, Medicap Clinic GmbH, Ulrichstein, Germany) at a rate of 25 to 40 ml/min. The column was loaded with plasma, the bound antibodies were eluted (Elution Solution ADAsorb, pH 2.2; Serag Wiessner GmbH, Naila, Germany), and the column was then recalibrated (multiPlus 2K, Fresenius Medical Care).

Immunoadsorption was performed on three consecutive days (days 1–3). In the event of relapse with elevated autoantibody levels, an additional three‐day IA cycle could be performed at any time during the study period.

**FIGURE 1:**
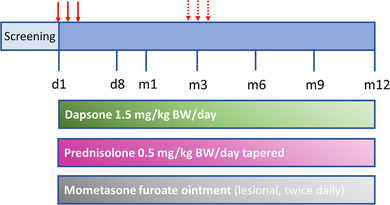
Treatment protocol. Immunoadsorption was performed on three consecutive days (red arrows). If required due to relapse, it was repeated at an individual time point (red dotted arrows). In addition, all patients received dapsone, prednisolone, and lesional mometasone furoate ointment. *Abbr*.: D, day; m, month; BW, body weight

As recommended by the German guidelines, patients received standard therapy with oral prednisolone (0.5 mg/kg BW per day, tapered), dapsone (1.5 mg/kg BW per day) and, deviating from the guideline, mometasone furoate ointment (lesional application, twice daily) (Figure 1).^15^ The dose of prednisolone was maintained until no new blisters develop for a period of 1 week. Prednisolone was then reduced by 25%. When no new lesions appeared, weekly tapering continued – initially by 25% (to 0.25 mg/kg BW per day), followed by 5 mg decrements until a dose of 10 mg per day was reached, and subsequently by 2.5 mg decrements until 5 mg per day. Subsequent dose reductions were performed at two‐week intervals to 2.5 mg per day, then to 2.5 mg every other day, followed by discontinuation. Dapsone 1.5 mg/kg BW per day was given in one morning dose until prednisolone was tapered off. After discontinuation of prednisolone and no new occurrence of blisters for 1 month, dapsone was reduced by 25 mg at monthly intervals and finally discontinued. Mometasone furoate ointment was initially applied to the affected skin areas twice daily and was omitted when no new lesions have appeared over a period of 1 week. In addition, blisters were opened sterile and, if necessary, treated with 0.5% pyoctanine solution.

Follow‐up visits included physical exams with assessment of the bullous pemphigoid disease area index (BPDAI) including Pruritus Score (0: no evidence of itch, 10: mild itch, 20: moderate itch, 30: severe itch), routine laboratory parameters and circulating anti‐BP180 NC16A IgG and anti‐BP230 IgG by ELISA (Euroimmun) as well as DLQI. Karnofsky Index was determined while screening and at the last study visit. Treatment outcomes were categorized into complete remission on therapy (healing of all lesions while on treatment), partial remission (healing of > 50% of lesions), and relapse (3 or more new lesions in the last month that do not heal within 1 week in a patient who has achieved complete remission before).

### Statistical analysis

The p‐values were determined using the Wilcoxon signed‐rank test. Statistical analyses were performed with GraphPad Prism (version 7; GraphPad Software, San Diego, CA, USA). The significance level was set at α = 0.05.

## RESULTS

### Clinical disease course

**FIGURE 2:**
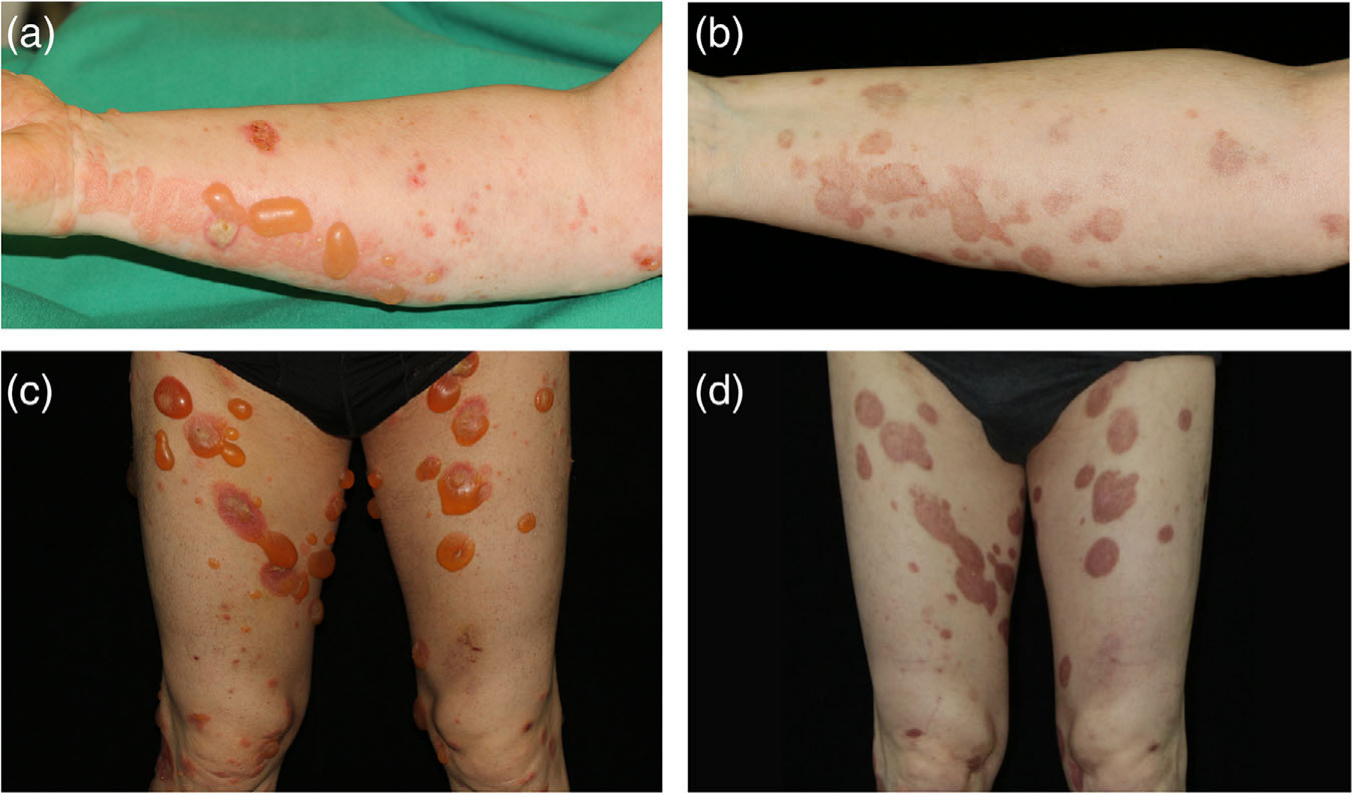
Clinical course. (a, c) Tense blisters on erythematous skin in patients 6 and 8 prior to the first cycle of immunoadsorption. (b, d) Re‐epithelialization with post‐inflammatory hyperpigmentation 4 weeks after the first cycle of immunoadsorption in the same patients.

**FIGURE 3:**
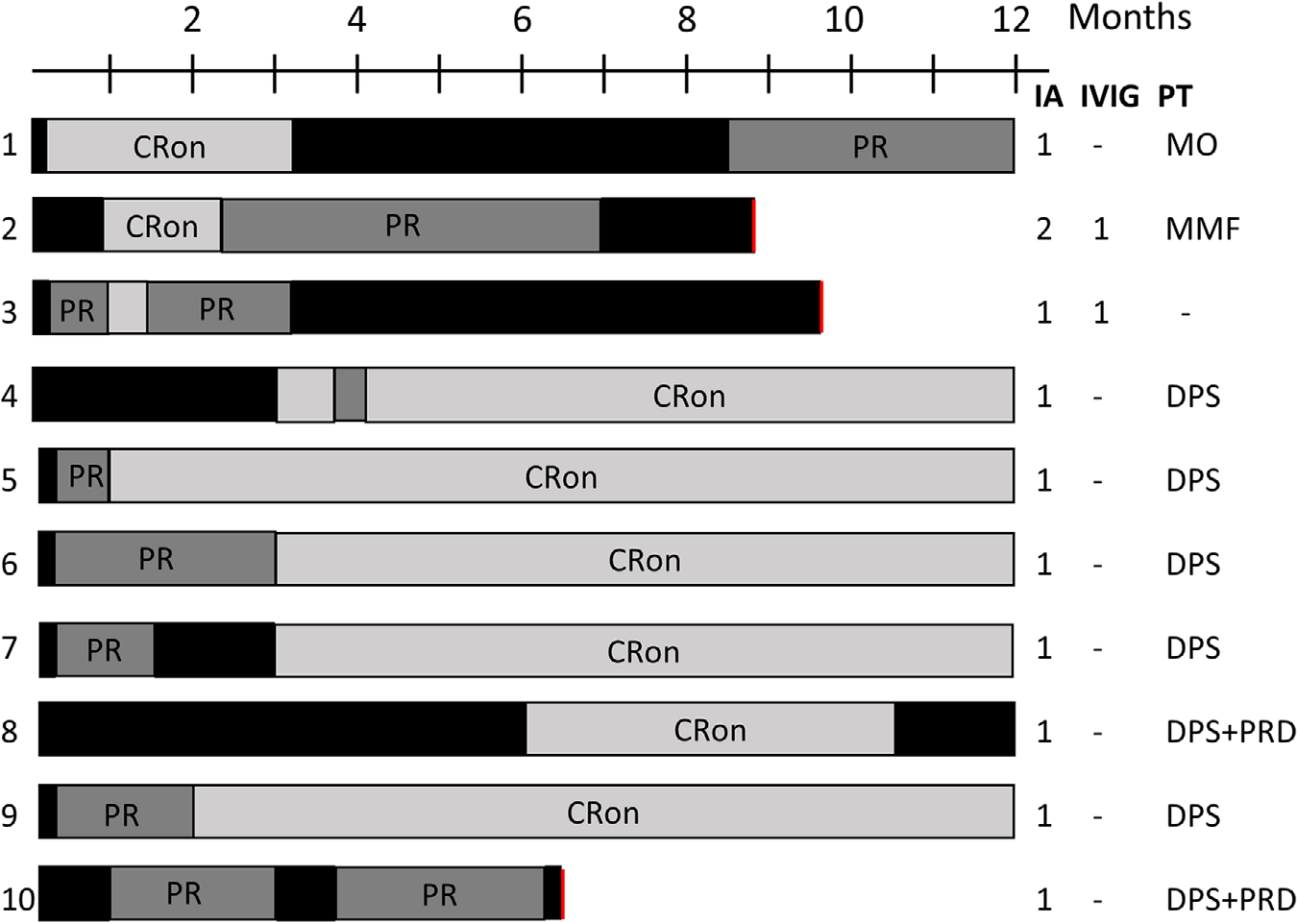
Disease courses and treatment details. Black box, active disease or relapse. *Abbr*.: PR, partial remission; CRon, complete remission on therapy; red bar, exclusion from study; IA, immunoadsorption cycle (3 days); IVIG, high‐dose intravenous immunoglobulin; PT, present therapy at study end; DPS, dapsone; MMF, mycophenolate mofetil; MO, mometasone furoate ointment; PRD, prednisolone

**FIGURE 4:**
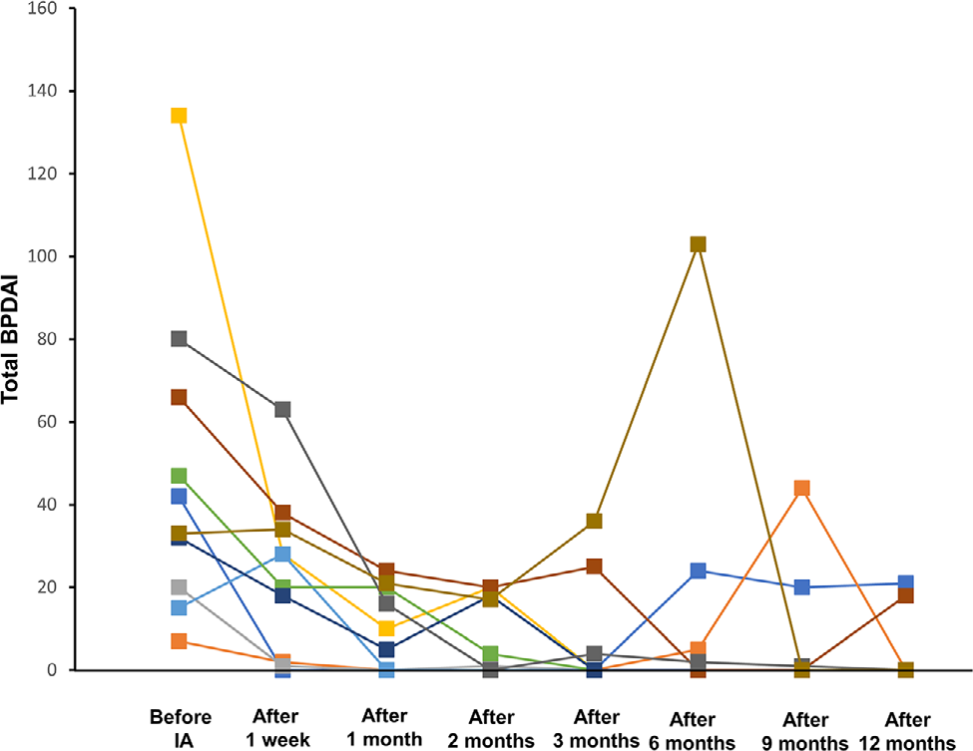
Total BPDAI values during the course of the disease. Each patient is represented by a specific color. *Abbr*.: IA, immunoadsorption; BPDAI, bullous pemphigoid disease area index

Within 2 months after IA, 50% of patients achieved complete remission on therapy, and within 6 months, the number increased to 90% (Figures 2, 3). Disease activity measured by total BPDAI has decreased significantly already 1 week after IA compared to baseline (p = 0.0137). At later time points – i.e., 1 (p = 0.002), 2 (p = 0.002), 3 (p = 0.0039), 6 (p = 0.0488), 9 (p = 0.0273), and 12 months (p = 0.002) after the initial IA – BPDAI values were also significantly reduced (Figure 4). Prednisolone was tapered and completely omitted after an average of 5.5 months. Within the follow‐up period of 12 months, six patients relapsed. According to the protocol, a second IA cycle could be performed in case of relapse, which was done in one patient (Patient 2). Seven of ten patients were followed up until the end of the study (12 months), two patients dropped out because of a second relapse (after 6 and 9 months, respectively) and one because of continued hospitalization for other reasons (after 9 months). None of the ten patients deceased during the follow‐up period of 1 year. Disease courses and BP medication are detailed in Figure 3. Quality of life has significantly improved in all patients 1 month after IA compared to baseline (p = 0.0039). In line, pruritus decreased significantly the week after IA from a score of 10.0 to 0 (p = 0.0156). All DLQI and pruritus scores are detailed in online supplementary Table S1.

### Comedication with prednisolone and dapsone

Prednisolone was initially given at a dose of 0.5 mg/kg BW per day and then tapered as described in the patients and methods section. In eight patients, prednisolone could be discontinued; three of these patients relapsed after prednisolone was completely discontinued, in one of them prednisolone was reintroduced. On average, prednisolone was discontinued after 5.5 months (range, 3 months to ongoing). All patients received less than 7.5 mg/day 6 months after initiation of therapy. Dapsone was continued in seven patients beyond the end of the study. In the remaining three patients, it was discontinued due to anemia/dyspnea, detailed in the section adverse events.

### Autoantibody levels

**FIGURE 5:**
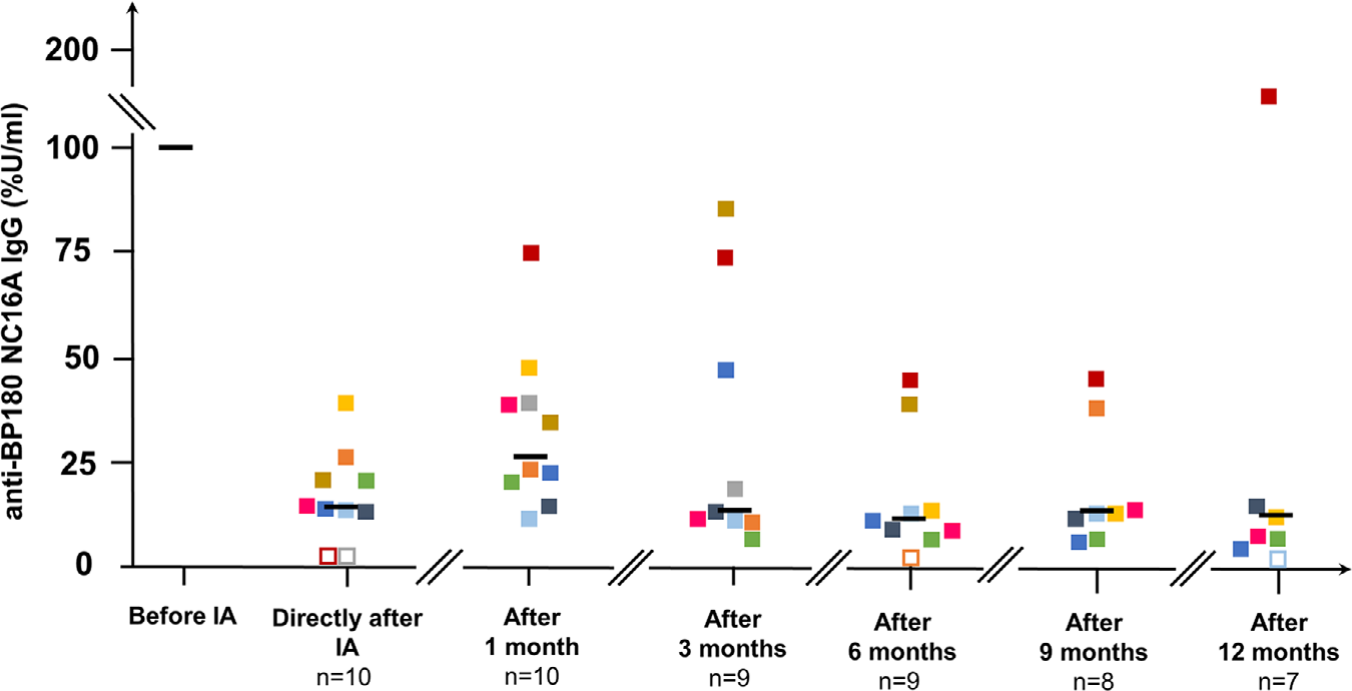
Serum levels of anti‐BP180 NC16A IgG as percentage of initial levels with median during the course of the disease. Positive values (> 20 U/ml) are shown by filled boxes, negative values by empty boxes. Each patient is represented by a specific color. Median anti‐BP180 IgG levels are indicated by black bars. *Abbr*.: IA, immunoadsorption

In all patients, serum levels of autoantibodies to BP180 decreased significantly after IA by an average of 89% from baseline compared to immediately after the third session of IA (p = 0.004), and by 73% 4 weeks later (p = 0.002). Also, 3, 6 and 9 months after IA, anti‐BP180 antibody levels were significantly lower compared to baseline (p = 0.004, p = 0.004, and p = 0.008, respectively). Patients 3 and 8 had no detectable anti‐BP180 serum IgG immediately after the 3^rd^ IA (Figure 5). Anti‐BP230 IgG serum levels decreased significantly 4 weeks (p = 0.03) and 6 months (p = 0.03) after IA. Anti‐BP180 and anti‐BP230 IgG serum levels are detailed in online supplementary Tables S2 and S3.

### Adverse events

A total of 56 AEs occurred during the 12‐month follow‐up (1 to 12 AEs/patient). The severity was classified due to the *Common Terminology Criteria for Adverse Events V3.0*. The majority of AEs were of grade 2 (50%), the other AEs were classified as grade 1 (34%), grade 3 (13%), and grade 4 (3%). Anemia and/or dyspnea were observed ten times and were mainly classified as grade 1 or 2 (90%). In Patient 1, dapsone was paused after the first episode of anemia and erythrocyte concentrates were given. When dapsone was restarted at a lower dose, anemia reoccurred shortly thereafter despite normal glucose 6‐phosphate dehydrogenase and dapsone was discontinued completely. In Patients 2 and 3, dapsone was discontinued because of anemia with hemoglobin levels below 8.6 g/dl despite normal glucose 6‐phosphate dehydrogenase and not restarted due to ongoing anemia. In Patients 5 and 7, dapsone was reduced, which then led to a normalization of hemoglobin levels.

Fifteen AEs were classified as severe AE, of which eight (angina pectoris, eczema, BP relapse, hypertensive crisis, and unclear infection) were possibly related to the study medication, while for seven (gout, bronchial carcinoma, norovirus infection, bursitis olecrani, pelvic ring fracture, intensive care treatment, fall) a relationship to the study medication seemed unlikely. The median interval between last day of IA and the occurrence of severe AEs was 84 days (range, 26–277 day). The majority of severe AEs (7(46%)) occurred in one patient (Patient 3). All AEs are detailed in online supplementary Table S4.

## DISCUSSION

Following the introduction of IA in 1969, this procedure has also been used in severe and/or refractory cases of AIBD including BP. So far, 35 patients with BP treated with adjuvant IA have been reported, most of them as case reports or small case series.^28^, ^29^, ^30^, ^31^, ^32^, ^33^, ^34^ No randomized controlled trial of BP subjected to IA is available. In 1997 Ino et al. treated the first two BP patients with the regenerable dextran sulfate cellulose column (Selesorb, Kanegafuchi Chemical, Osaka, Japan) in combination with corticosteroids and corticosteroids plus dapsone, respectively.^31^ One patient received six apheresis treatments over 2 weeks, followed by clinical remission. In the second patient, IA was performed four times during a two‐week period and was repeated four times when 6 weeks after the first treatments skin lesions relapsed. Complete remission was not achieved in any of them. Herrero‐Gonzáles et al. reported IA in two BP patients refractory to first‐line treatment using a single‐use, non‐regenerable tryptophan‐linked matrix Immusorba TR‐350^®^ (Asahi Medical, Tokyo, Japan).^32^ Two IAs on consecutive days were performed, in one patient one cycle of IVIG was applied to prevent infections. Both patients achieved complete remission on therapy after 6 weeks. In 2012, Müller et al. described a BP patient unresponsive to second‐line treatment with IVIG.^34^ Two cycles of IA on three consecutive days with an interval of 14 days with the regenerable, reusable protein A column Immunosorba^®^ (Fresenius Medical Care) was employed and led to partial remission on therapy. In the case series by Kasperkiewicz et al. including seven BP patients, IA was applied on three consecutive days (Immunosorba^®^, Fresenius Medical Care) in combination with prednisolone 0.25 mg/kg BW per day, dapsone 1.0–1.5 mg/kg BW per day, and clobetasol propionate 0.05% ointment twice daily.^29^ All patients achieved complete remission on therapy 1 to 3 months after IA and six of the seven patients showed long‐term response. Using the same adsorber, in three patients IA was performed 7–10 times over a period of 4–9 weeks in combination with rituximab and conventional immunosuppression by Kolesnik et al.^33^ Two patients showed complete remission, whereas one patient only achieved partial remission.

The largest case series of IA in BP to date, published by Hübner et al., included 20 patients.^28^ IA was performed on three consecutive days, i.e., one cycle (Immunosorba^®^, Fresenius Medical Care), in addition to dapsone, prednisolone, and topical clobetasol propionate ointment. Two of 18 patients required additional cycles of IA. One of the patients received one, and another three additional cycles in an interval of 2 weeks. All patients showed a rapid and sustained treatment response and anti‐BP180 serum IgG decreased to 26% of baseline after 1 month.^28^ In contrast to latter study, in the present study, all patients were treated prospectively according to a standardized protocol.

We treated ten BP patients with one cycle of IA on three consecutive days, as well as dapsone (1.5 mg/kg/day), prednisolone (0.5 mg/kg/day), and lesional application of mometasone furoate ointment twice daily. Prednisolone was tapered relatively fast. The favorable results of the previous case series, the clear pathogenic role of autoantibodies in BP, and the need of new therapeutic options for severe and/or refractory BP patients provided the rational for our study. In particular, since corticosteroids – still the cornerstone of BP treatment – are associated with serious adverse events and increased mortality,^35^ therapies that can reduce the cumulative corticosteroid dose and, consequently, the number and severity of adverse events are urgently needed. Along this line, a recent randomized controlled trial investigating adjuvant IA in pemphigus vulgaris and pemphigus foliaceus demonstrated a significant reduction in the cumulative corticosteroid dose in the IA plus best medical treatment group compared with patients receiving best medical treatment alone.^27^

In general, reusable and single‐use devices as well as regenerable and non‐regenerable columns can be differentiated. Non‐regenerable devices contain the ligands phenylalanine, tryptophan, dextran sulfate, and hydrophobic amino acid and can be applied in a single session until their capacity is exhausted. In contrast, regenerable adsorbers are used in pairs. As such, while one column is regenerated, the other can be loaded with plasma, thus a continuous apheresis process is possible. Protein A, the synthetic peptide PGAM146, or sheep antibodies directed against human Ig are used as ligands in these regenerable reusable adsorbers which can be reused for up to 10–20 procedures in the same patient.^36^

Compared to the study by Hübner et al., where a reusable regenerable protein A column (Immunosorba^®^, Fresenius Medical Care) was employed,^28^ the current study applied a single‐use but regenerable protein A column (Ligasorb^®^, Fresenius Medical Care). As such, one column was applied for each IA procedure that reduced the costs for the column material by 25% compared to the use of the Immunosorba^®^ column pair in our previous study.^28^

In the present study, within 2 months after IA, 50% of patients showed complete remission on therapy which is comparable with the results by Hübner et al. who showed complete remission in 42% of patients 1 month after IA.^28^ In latter case series, IA was particularly successful in patients refractory to previous treatment, while in our study, both refractory and treatment‐naive severely affected patients responded accordingly.

In contrast to the report by Hübner et al., the present study also quantified pruritus and life quality during the disease course. Remarkably, life quality improved significantly from 19.1 ± 8.0 (mean ± standard deviation [SD]) to 6.8 ± 6.3 already 1 month after IA (p = 0.0039). Likewise, pruritus decreased significantly as early as one week after IA, from a median score of 10.0 to 0 (p = 0.0156).

In the present cohort, all patients had at least one AE (in total 56 AE), which is higher than in the study by Hübner et al. which reported at least one AE in only 65% of patients. Ten of the 56 AE in the present study were anemia/dyspnea, which is a common side effect of dapsone. In three of five patients, dapsone had to be stopped permanently, while in the two others, dose reduction of dapsone led to an increase of hemoglobin. All in all, 15 AE in five patients were classified as severe (grade 3 or 4), of which eight were possibly related to the study medication, while in seven, a relationship seemed unlikely. The median interval between the last day of IA and the occurrence of the severe AE was 84 days, the earliest AE occurring 26 days after the last IA. Severe AE possibly related to IA were angina pectoris, eczema, BP relapse, hypertensive crisis, and unclear infection in four patients arising 35, 28, 26, 50, 71, 223, 28, and 81 days after the last IA, respectively. Taken together, it cannot be excluded that some severe AE were related to the IA procedure. In our opinion, the considerable interval between their occurrences does not support a causal relationship. Of note, with the exception of an unclear infection in Patient 3 12 weeks after the last IA, no infection was recorded within the cohort. Infections have previously been related to IA, mostly in patients with central venous access.^27^ In the present study, in all patients, a central line was used.

Of note, the majority of severe AE (46%) occurred in a single patient. This patient had multiple co‐morbidities including coronary heart disease (what was not considered a severe cardiovascular disease at study inclusion because it was asymptomatic) and renal failure, and was 10 years older than the mean age of the cohort. So far, increased age has not yet been related to an increased rate of severe AEs in patients receiving IA.^22^, ^36^ In fact, Hübner et al. reported a 94‐year old female with BP that developed anemia as only AE. Of interest, none of our patients died during the follow‐up period of 1 year although the one‐year mortality in BP is 20–30% and about 3–5‐fold higher compared to sex‐ and age‐matched controls.^37^, ^38^, ^39^ One may speculate that the low mortality in our cohort was attributed to the potentially corticosteroid‐sparing regimen allowing a more rapid prednisolone tapering. This hypothesis is supported by the observation of a one‐year mortality of 16% in a multicenter observational study with 198 BP patients that received prednisolone alone at an initial dose of 0.5 mg/kg BW per day.^40^

In all our patients, serum levels of IgG autoantibodies to BP180 dropped significantly after IA, by an average of 85% from baseline immediately after the third consecutive session of IA, and by 68% 4 weeks later. This is comparable with the case series of Hübner et al. that observed an initial decrease of anti‐BP180 IgG antibodies by 92% immediately after the third consecutive session of IA and by 74% 4 weeks later. Collectively, we hypothesize that IA, by rapidly reducing circulating autoantibody levels, helps to decrease disease activity in the early treatment phase, which subsequently allows for a faster corticosteroid taper. Because of the severe side effects of corticosteroids this is a great advantage for our patients.

A limitation of our study is the lack of a control group which would have meant a much larger study size of about 100 patients as previously estimated in the IA‐Pem study for pemphigus.^27^ Another limitation is the use of IA as adjuvant treatment in combination with a regimen recommended for moderate/ severe BP by national and international guidelines.^14^, ^41^

In summary, our prospective non‐controlled monocenter study showed that IA is a relatively safe and potentially effective adjuvant therapy for patients with severe and/or refractory BP. Since a randomized controlled trial that would provide high‐level evidence on the value of IA in BP is unlikely to be conducted, the data presented here may remain the highest available evidence regarding the safety and efficacy of IA in this disease.

## FUNDING INFORMATION

The work was supported by Fresenius Medical Care.

## CONFLICT OF INTEREST STATEMENT

E.S. has received honoraria for lectures and travel grants from Fresenius Medical Care. E.S. has a scientific cooperation with Fresenius Medical Care. N.v.B. has received honoraria for lectures from Fresenius Medical Care.

## Supporting information



Supplementary information
