# Supplementary material for: Adjuvant therapy of severe and/or refractory bullous pemphigoid with immunoadsorption – A prospective monocenter pilot study
Source: J Dtsch Dermatol Ges. 2025 Nov 2;24(4):472–9. doi: 10.1111/ddg.15909 (PMC13059066; doi:10.1111/ddg.15909)
Supplement: Supplementary file 1 — Supplementary information [file DDG-24-472-s001.docx]

Supplementary material

Table S1.

Patient DLQI DLQI DLQI DLQI DLQI Pruritus Pruritus Pruritus Pruritus Pruritus Pruritus Pruritus Pruritus

no. baseline 1 month 3 months 6 months 12 months baseline day 8 1 month 2 months 3 months 6 months 9 months 12 months

1 12 0 0 2 0 30 na 0 na 0 20 10 10

2 13 1 0 0 ex 0 0 0 0 0 0 0 ex

3 14 10 23 na ex 10 0 0 0 na na na ex

4 27 1 1 2 0 10 0 10 10 0 0 0 0

5 21 7 3 5 0 0 0 na 20 10 10 0 0

6 27 19 9 4 0 30 10 10 0 10 10 10 10

7 2 5 5 0 0 10 0 0 0 0 0 0 0

8 26 17 10 5 26 10 0 10 20 20 10 10 0

9 23 5 7 7 9 30 10 10 10 0 0 0 0

10 26 3 7 22 ex 30 10 10 0 10 30 ex ex

*Abbr.:* Patient no., patient number; DLQI, Dermatology Life Quality Index; na, not available; ex, exclusion from study.

Table S2.

Patient BP180 BP180 BP180 BP180 BP180 BP180 BP180 BP180 BP180 BP180

no. Screening before 1^st^ IA after 1^st^ IA day 8 1 month 2 months 3 months 6 months 9 months 12 months

1 549 1,482 148 250 124 na 122 186 169 7

2 179 450 128 144 104 74 45 0 162 na

3 645 377 0 154 155 132 73 na na ex

4 400 364 139 373 170 111 na 60 42 38

5 387 618 61 169 87 58 77 37 48 87

6 160 797 142 170 130 73 32 40 34 33

7 208 1,881 208 na 379 1,046 884 171 50 29

8 180 100 0 56 75 72 71 45 43 154

9 329 1,072 127 597 428 173 121 77 134 115

10 651 382 73 106 122 202 326 143 ex ex

*Abbr.:* BP180, anti-BP180 NC16A IgG ELISA levels (U/ml); no., number; na, not available; ex, exclusion from study.

Table S3.

Patient BP230 BP230 BP230 BP230 BP230 BP230 BP230 BP230 BP230 BP230

no. Screening before 1^st^ IA after 1^st^ IA day 8 1 month 2 months 3 months 6 months 9 months 12 months

1 109 263 183 157 114 na 104 176 537 398

2 140 152 134 127 83 50 31 0 20 ex

3 0 0 0 0 0 0 na na na ex

4 86 70 0 35 66 0 na 0 0 na

5 21 0 0 0 0 0 0 na 0 0

6 0 46 0 33 0 0 0 0 0 0

7 119 325 84 na 163 128 151 77 na na

8 na na na na 0 0 0 0 0 0

9 140 164 na 142 137 100 64 91 59 67

10 na na 0 na na na na na ex ex

*Abbr.:* BP230, anti-BP230 IgG ELISA levels /U/ml); no., patient number; na, not available; ex, exclusion from study.

Table S4.

Patient AEs

no. (days after IA)

1 anaemia (171), urinary tract infection (184), anaemia (277), pelvic ring fracture (277), fall (534)

2 eczema (28), angina pectoris (35), increased liver enzymes (35), anaemia (35), increased liver enzymes (172), anaemia (172), BP replapse (223), BP relapse (26*)

3 anaemia (7), intertrigo (71), BP relapse (71), unclear infection (81), bursitis olecrani (84), gouty arthritis (96), carpal-tunnel syndrome (99), urinary tract infection (114), bronchial carcinoma (118), norovirus infection (128), clostridium difficile colitis (128), intensive care treatment (145)

4 acneiforme rash (110)

5 anaemia (374)

6 nausea/vomiting (0), arterial hypertension (0), dyspnoea (19), hypertensive deregulation (54), dyspnoea (82)

7 anaemia (26), lactate dehydrogenase increase (26), BP relapse (50)

8 nausea (0), vertigo (0), blue discoloration on the toe (32), BP relapse (82), acne (139), BP relapse (317), BP relapse (362)

9 lower leg edema (4), tinea (172), hordeolum (285)

10 fatigue (0), tinea (7), supraventricular tachycardia (28), pericardial effusion (28), hypertensive deregulation (28), rash on legs (49), dyspnoea (54), arterial hypertension (28), BP relapse (82), ear noise (90), BP relapse (185)

*Abbr.:* no., number; AE, adverse event; IA, immunoadsorption; BP, bullous pemphigoid.
